# Supplementary material for: MiR-196a Promotes Lipid Deposition in Goat Intramuscular Preadipocytes by Targeting MAP3K1 and Activating PI3K-Akt Pathway
Source: Cells. 2024 Aug 30;13(17):1459. doi: 10.3390/cells13171459 (PMC11394330; doi:10.3390/cells13171459)
Supplement: Supplementary file 1 [file cells-13-01459-s001.zip › Supplementary Figures.pdf]

Supplementary Material

MiR-196a Promotes Lipid Deposition in Goat Intramuscular Preadipocytes by Targeting *MAP3K1* and Activating PI3K-Akt Pathway

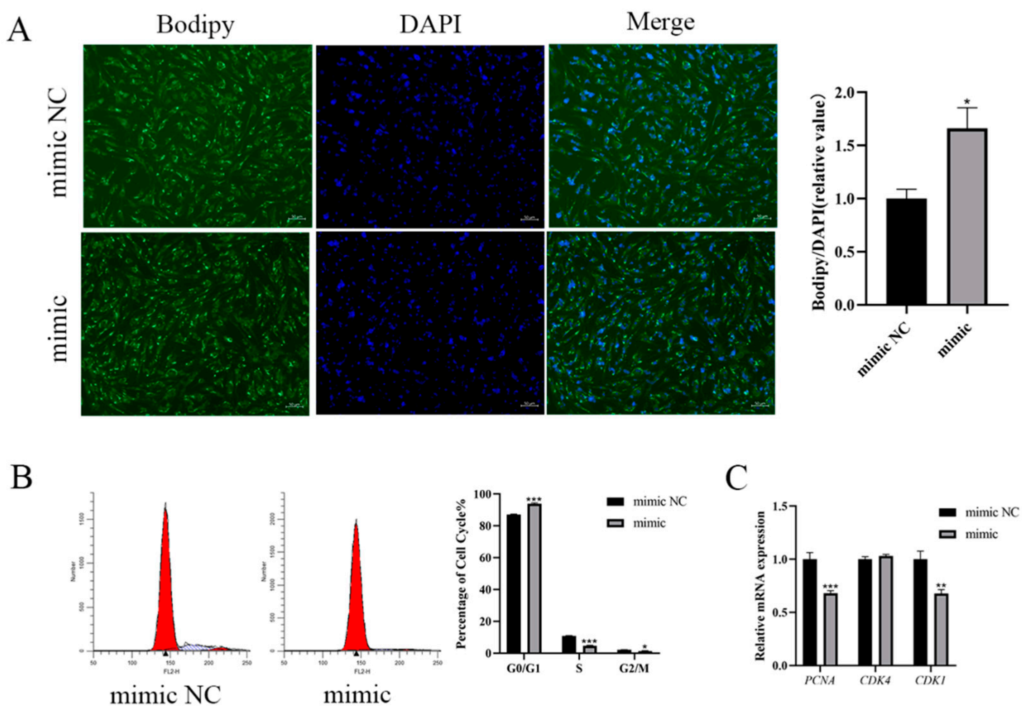

Figure S1. Overexpression of miR-196a promotes adipogenesis and inhibits cell proliferation in goat preadipocytes. (**A**) Bodipy staining and quantification of lipid droplets after overexpression of miR-196a (day 2 after adipogenic differentiation, 100×). (**B**) The cell cycle of preadipocytes treated with miR-196a mimics. (**C**) The relative expression levels of proliferation-related genes after miR-196a mimic treatment. The data are expressed as means ± SEM. * $p < 0.05$, ** $p < 0.01$, and *** $p < 0.001$.

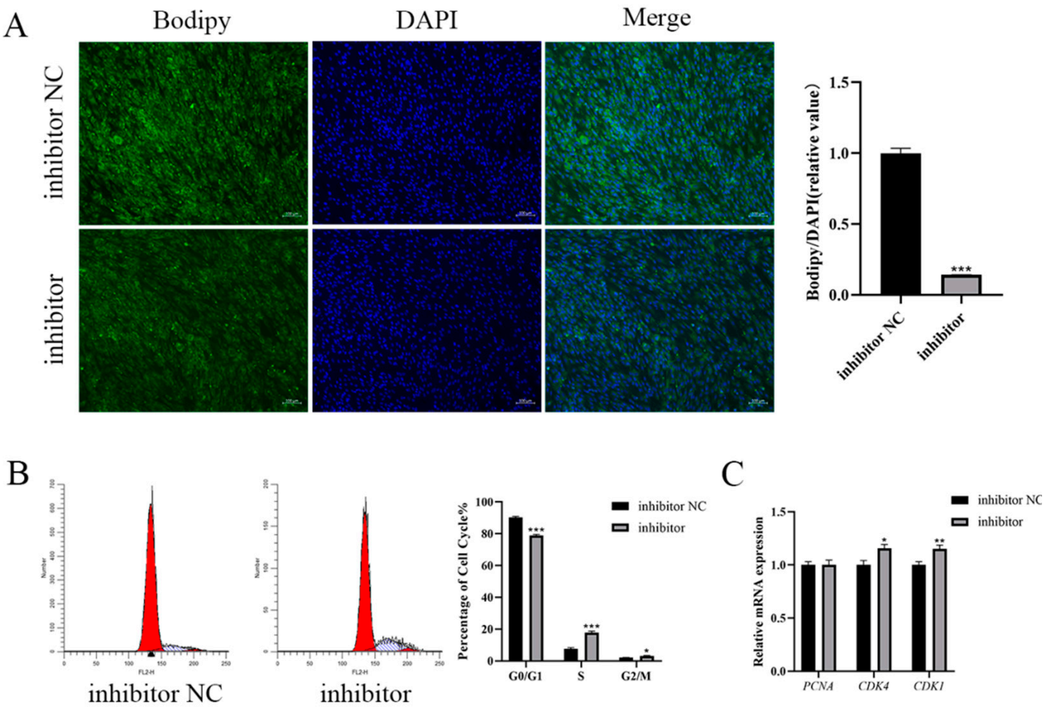

Figure S2. Interference with miR-196a inhibits adipogenesis and promotes cell proliferation. (**A**) Bodipy staining and quantification of lipid droplets after miR-196a interference (day 2 after adipogenic differentiation, 100×). (**B**) Detection of cell cycle of preadipocytes treated with miR-196a inhibitor. (**C**) The relative expression levels of proliferation-related genes after miR-196a inhibitor treatment. The data are expressed as means ± SEM. * $p < 0.05$, ** $p < 0.01$, and *** $p < 0.001$.

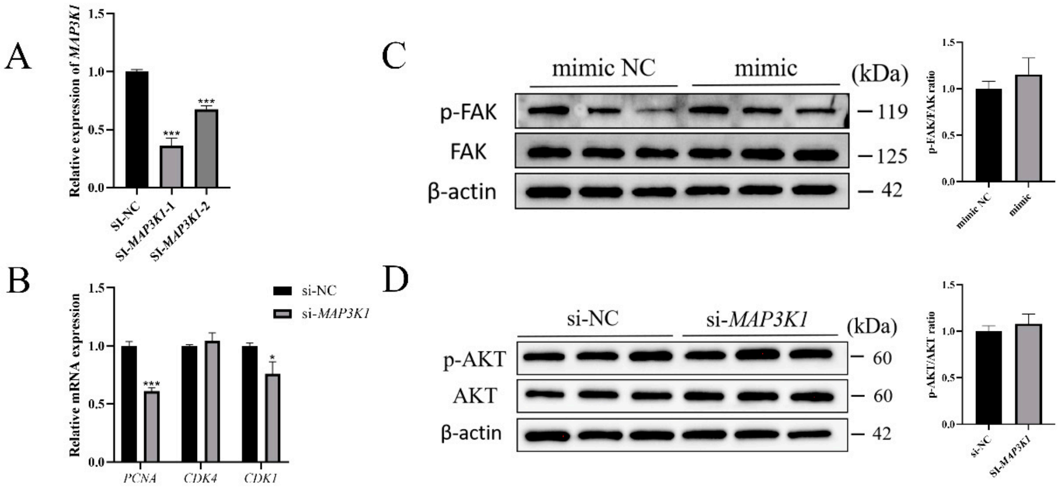

Figure S3. miR-196a and its target gene *MAP3K1* affect FAK and PI3K-Akt signaling pathways. (**A**) *MAP3K1* interference efficiency detection of two si-*MAP3K1*. (**B**) The relative expression levels of proliferation-related genes after si-*MAP3K1* treatment. (**C**) WB was used to detect the expression of phospho-FAK (p-FAK) and total FAK proteins in cells treated with miR-196a mimic. (**D**) WB was used

to detect the expression of phospho-AKT (p-AKT-S473) and total AKT proteins in si-*MAP3K1* and si-NC treated cells. The data are expressed as means ± SEM. * $p < 0.05$, and *** $p < 0.001$.
